# Navigating the complexities of mobile medical app development from idea to launch, a guide for clinicians and biomedical researchers

**DOI:** 10.1186/s12916-023-02833-7

**Published:** 2023-03-23

**Authors:** Robert G. Mannino, Santiago J. Arconada Alvarez, Morgan Greenleaf, Maren Parsell, Comfort Mwalija, Wilbur A. Lam

**Affiliations:** 1 Georgia Clinical Translational Science Alliance, Atlanta, USA; 2 Emory University School of Medicine, Atlanta, USA; 3 School of Interactive Computing, Georgia Institute of Technology, Atlanta, USA; 4 Global Health Informatics Institute, Lilongwe, Malawi; 5 Wallace H. Coulter Department of Biomedical Engineering, Emory University and Georgia Institute of Technology, Atlanta, USA; 6 Aflac Cancer and Blood Disorders Center of Children’s Healthcare of Atlanta, Atlanta, USA

**Keywords:** Smartphone app development, Design thinking, Human-centered design, Healthcare technology

## Abstract

With today’s pace of rapid technological advancement, many patient issues in modern medicine are increasingly solvable by mobile app solutions, which also have the potential to transform how clinical research is conducted. However, many critical challenges in the app development process impede bringing these translational technologies to patients, caused in large part by the lack of knowledge among clinicians and biomedical researchers of “what it takes” to design, develop, and maintain a successful medical app. Indeed, problems requiring mobile app solutions are often nuanced, requiring more than just clinical expertise, and issues such as the cost and effort required to develop and maintain a well-designed, sustainable, and scalable mobile app are frequently underestimated. To bridge this skill set gap, we established an academic unit of designers, software engineers, and scientists that leverage human-centered design methodologies and multi-disciplinary collaboration to develop clinically viable smartphone apps. In this report, we discuss major misconceptions clinicians and biomedical researchers often hold regarding medical app development, the steps we took to establish this unit to address these issues and the best practices and lessons learned from successfully ideating, developing, and launching medical apps. Overall, this report will serve as a blueprint for clinicians and biomedical researchers looking to better benefit their patients or colleagues via medical mobile apps.

## Background

Smartphones are driving a technological and patient-centric revolution in healthcare [1, 2]. The technological sophistication and ubiquitous use of modern smartphones are increasingly enabling app-based solutions to clinical problems [3–5]. For example, smartphones are commonly used to enhance the patient-physician relationship by allowing patients to send images to their physician remotely for condition tracking and monitoring [6–8]. This increase in mobile medical solutions is apparent in the literature, where an exponential increase in academic publications available on PubMed can be seen (> 5000 manuscripts published in 2021 alone) [9]. However, there are currently major systemic barriers in place in the field of academic medicine that hinder the development of more sophisticated solutions [10].

## Main text

### Misconceptions in mobile medical app development

**Fig. 1:**
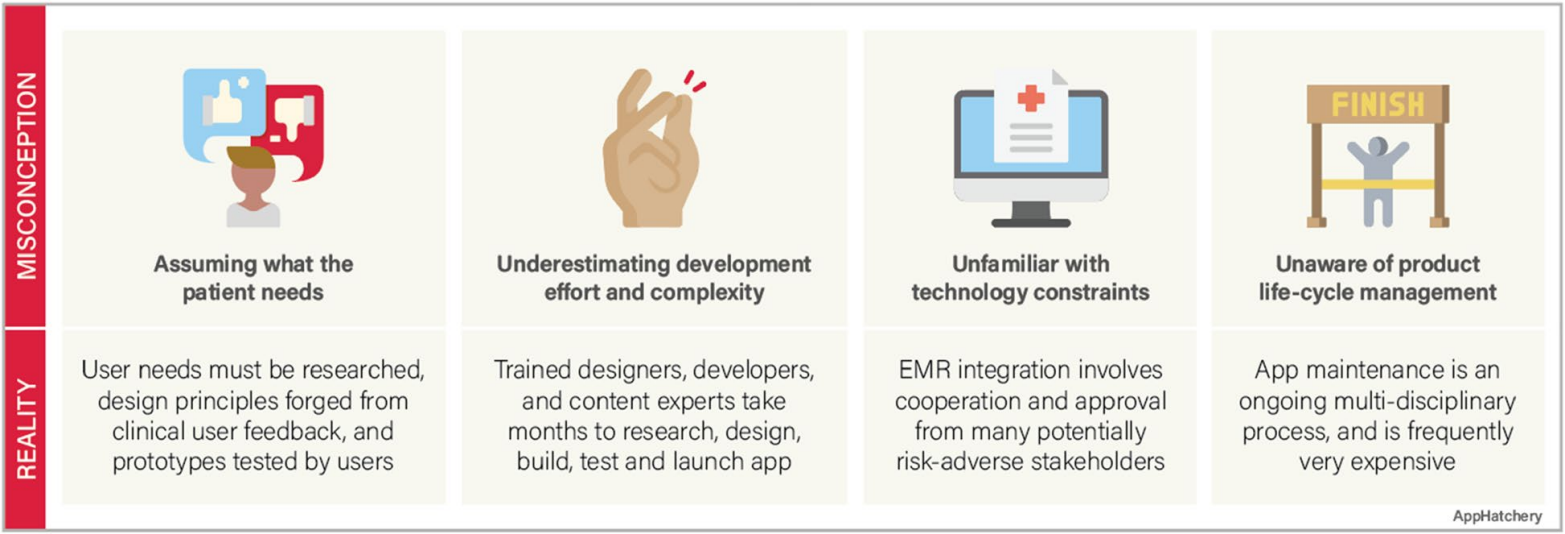
Common misconceptions of medical mobile app development

Despite the advances in medical technology and the increased availability of mobile app solutions in medicine, there are major misconceptions among clinicians involved in building a mobile app (Fig. 1).

#### Misconception #1—the investigator fully understands the problem they are trying to solve and the users they intend to help

##### Reality

Before the development process can start, the competitive landscape should be assessed to confirm that there is not already an identical or similar goal solution. Next, the intended users must be interviewed to ensure that the solution solves a relevant problem for them. This user research should be incorporated throughout the development process to assure that the users are continually being considered. An effective team must also be formed with the required expertise to handle every phase of the development lifecycle, from clinical problem identification, user research, design, development, and product release. Finally, a strategy must be developed to market the product so that users discover and adopt the app.

#### Misconception #2—app development does not require much effort

Reality

##### Reality

A common phrase we hear among our clinical colleagues when we are approached with an idea for a mobile medical solution is “…and I’d like to build an app.” This statement can be interpreted as, “I would like to hire a contractor to turn my idea into an app.” This line of reasoning underestimates the investigator commitment required to turn an idea into a mobile app. Throughout the development process, the investigator must play an active role in the development team. This process frequently takes months of user research, design, development, and testing in an iterative fashion.

#### Misconception #3—the app should easily integrate into the electronic health records (EHR) system

##### Reality

The electronic medical records in the USA and many countries are highly fragmented, with individual hospital systems using their own 3rd party vendors to manage their records [11, 12]. Accessing a single institution’s EHR requires multiple levels of approvals at the principal investigator and administrative levels and then requires significant technical collaboration with the institution’s information technology department to connect. While Fast Healthcare Interoperability Resources (FHIR) standard has been adopted to facilitate a consistent format of healthcare information for simple transmission and sharing of data, the lack of a consistent EHR framework significantly impacts the ability of a 3rd party app to integrate into the EHR’s of a geographically diverse user base [13].

#### Misconception #4—app development is a single-step “one and done” process

##### Reality

The development process is not complete when the app is launched. A continuity plan must be in place to maintain and update the app throughout its lifecycle. Technology changes rapidly, operating systems update, and smartphone manufacturers develop and release multiple new phone models annually. An app must be maintained to adapt to these advancements. The request to “build an app” does not accurately convey that app development is a process that does not end with an app launch.

These misconceptions underscore a clear need for a liaison between clinicians and the app development community to facilitate the development of robust, maintainable, mobile apps. To address these misconceptions, we set up an academic sub-unit within the Georgia Clinical and Translational Science Alliance (Georgia CTSA), which we call the AppHatchery, with the goal of supporting clinical investigators in their endeavors to create and clinically translate their mobile medical app ideas. Care was taken to structure the AppHatchery to enable this end goal, with a mix of relevant expertise and experience. This initiative is led by a physician scientist who runs a laboratory that has a proven track record of developing and publishing medical apps as well as conducting clinical research using apps. We hired a product manager from the industry with expertise in managing mobile app development teams for a large organization. We have a designer on the team who specializes in user research and design thinking. Finally, we have multiple full-time developers to carry out mobile development work. We structured the group this way to ensure that we had members of the team with expertise in each phase of medical app development, specifically (1) clinical research expertise, (2) design, and (3) mobile app development. Given these diverse skillsets with every team member having some exposure to both clinical science and app development, we have created the perfect team to bridge the skillset gap between clinicians and software developers and develop mobile medical apps. Furthermore, the existence of team members fully dedicated to this initiative allows us to maintain continuity throughout each stage of the project at hand.

We first rely on internal marketing for project solicitations. When a project is onboarded, we perform a multi-disciplinary research process where we identify and explore the clinical and practical need for the given solution and its place within the current regulatory landscape and market. We use a combination of design thinking (DT) and human-centered design (HCD) framework to tackle app development challenges [14] from ideation through delivery. The design research process begins by conducting a deep dive into the problem as it pertains to end users and key stakeholders. Target users are interviewed to better understand their challenges, and we identify and/or verify the important features and aspects of the proposed app and get feedback on the design requirements that our research has identified. Simultaneously, we begin working with the principal investigator (PI) to craft the research study that will determine the efficacy of the tool we develop. Next, we begin the app development process, beginning with the design of wireframes and culminating with software development, where we conduct user research throughout the process to iteratively improve the app. We finally transition to launch and support, where we work with the principal investigator to release the app and support the clinical validation study. In this manuscript, we will describe the lessons learned from our journey and make recommendations for how other medical app developers should operate to successfully develop and translate clinical app ideas.

### App development principles and structure

#### Design approach

**Fig. 2:**
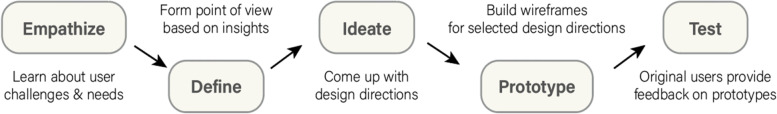
Design thinking framework

What makes our approach to building digital tools unique in the clinical space is our emphasis on both DT and HCD (Fig. 2). We use DT to focus on understanding who the users are, uncovering their needs, and empathizing with their situation first before kicking off the product design and development process [15]. In addition, we strategically consider user needs in relation to clinical objectives to form design recommendations [16, 17]. We then kick off the development phase which incorporates HCD fundamentals, bringing the end user’s voice and opinions into the development process. Collaborative strategies are noted as vital to sustaining growth in mobile health applications [18]. HCD has been demonstrated to be an effective innovation tool in the digital clinical and health space where a focus on end users (patients, clinicians, or others) during the design process improves adoption rates [19–21] as well as patient engagement and satisfaction [22].

A recent narrative review of 82 health innovation papers mentioning HCD found most (70%) discussed using an HCD approach and all included a focus on users’ needs and the participatory and iterative nature of the design process [14]. More salient limitations to the HCD approach include sampling bias and small sample size as users must opt into the research to participate in the design process and are not necessarily the profile of users who may need the app the most. This may lead to a misalignment of the design with the intended user’s needs. Finally, few apps have been tested in randomized controlled trials, and so limited evidence is available on the long-term benefits of app products arising from HCD approaches [23]. Specifically, in using HCD, there are instances where the approach did not lead to significant improvement in the intervention outcomes. For example, Gallagher et al. conducted a study using a game-based app that was co-designed to improve cardiovascular risk factors and lifestyle behaviors and found no improvement after the intervention [24]. Similarly, Haque et al. evaluated a user-centered mHealth app in promoting physical activity and found very low compliance at the end of the study which limits the generalizability of the results [25]. Furthermore, alternatives to DT and HCD such as user experience (UX) design frequently focus on the way users interact with apps and strive to provide the most convenient use for the people who actually use the app, rather than the ideal users that HCD and DT strive for [26]. In light of these findings, it is important to note that HCD methodology allows for subjectivity in terms of which aspects of user experience to prioritize, which can result in varied outcomes. Additionally, as previously discussed in the literature, HCD is susceptible to sampling bias, which can impact the apparent effectiveness of an intervention. Thus, it is crucial to consider both the strengths, limitations, and alternatives to DT and HCD and to employ a multi-disciplinary approach that considers various perspectives and factors in the design and evaluation process. This design strategy accurately defines the user, their needs, the problem most important to solve for that user, how those needs intersect with research goals, and which design directions are most effective at maintaining engagement and delivering results. It provides the foundation for the team who then focuses on the users and their needs throughout the app project development process.

#### Building a team

A development team must be created to begin the development process. A software development team has three distinct yet overlapping roles, the product manager, the designer, and the developer. In addition to these key roles, multiple supporting roles may be created for handling regulatory requirements necessary in medical app development.

##### Product manager

The product manager is responsible for the overall strategy of the mobile app. This includes the macro strategy of why and how to build the app in the first place as well as a more detailed strategy about which features to build, what order to build these features, and how to measure if the features are a success. The product manager is also responsible for creating the requirements in sufficient detail so the remainder of the team can execute their jobs effectively. These might be technical requirements, design requirements, or requirements from the clinical team. The product manager is also responsible for the lifecycle of the mobile app. This includes releasing the app through testing platforms, releasing the app publicly, working through approval processes at the institution, and maintaining the app’s public information on the app stores.

The product manager is responsible for ensuring the app meets guidelines. This is important especially for medical apps that may need to comply with the Health Insurance Portability and Accountability Act of 1996 (HIPAA) [27]. In our experience, projects are hindered at the beginning stages by focusing on these compliance issues instead of focusing on the core value of the mobile app. Compliance needs to be addressed at the beginning of a project when architecture is being designed and before the app is released publicly to ensure it meets all the required standards for privacy and security. The product manager is responsible for analytics and measuring the mobile app experience. These analytics are tied to the goals and objectives of the app (e.g., reducing missed follow-up visits) but also user experience metrics such as app crashes, screen views, and user interactions.

##### Software developer

The developer is responsible for writing the software code that will control the mobile app. This role is critical as there will be no software created without team members who can code the mobile app.

##### Designer

The designer is the empathizer in chief; it is their responsibility to always be the voice of the user throughout the app development process. This is a critical role as academic medical apps are often conceived without direct consultation of patients or users.

##### Other team roles

Developing mobile apps within an academic medical center requires knowledge of how Institutional Review Boards (IRB) review requests to conduct clinical research. It is useful to have an IRB expert on the team to answer nuanced questions about data collection and privacy that may differ from IRB to IRB and can be unique to clinical research involving mobile apps. While not critical in the early clinical research phases, it can be useful to have a regulatory expert on the team who is familiar with the relevant regulation, specifically around HIPAA and managing protected health information (PHI).

#### Full time vs part-time staff

Many academic medical centers operate on a shared time model where the staff dedicate a portion of their time to projects. While this can work, we have found it is better to have full-time staff dedicated to the development of mobile apps. Likewise, many software development projects are outsourced to other groups. While this can work, we have found it is best to work cohesively as a team throughout the development process to streamline communication and align expectations throughout.

### Applying the app development process

#### Competitive and regulatory landscape survey and literature review

**Fig. 3:**
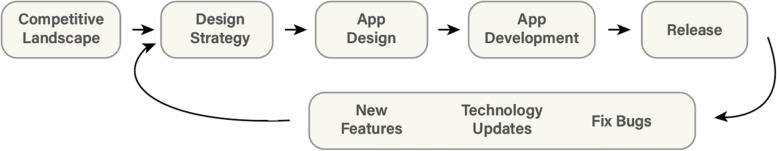
App project development process

Before accepting any new project, it is important to meet with the PI to understand their clinical and application vision before conducting an extensive search of the major app stores to see what similar apps currently exist, as well as related platforms, tools, books, and/or educational materials in the space. After the search is completed, the list of these competing resources should then be presented to the PI to discuss how or if the value proposition of their idea merits the effort to build a homegrown solution. If the concept meets the acceptance criteria set by the team (i.e., is novel, addresses a real need, and the development team has capacity and knowledge), the project can proceed (Fig. 3).

When researching and deciding the intended use case of the app, research into the regulatory pathway required to market the product must be conducted. In the United States of America (USA), the US Food and Drug Administration (FDA) approves medical devices for sale in the US market. FDA guidance regarding software as a medical device (which includes medical apps) is governed specifically by the 21st Century Cures Act [28]. Section 3060 of this legislation defines what software the FDA considers to be a medical device that requires regulatory approval via the 510(k) premarket notification pathway, versus lifestyle tools that can immediately enter the market [28]. Ultimately, the distinction is determined based on the function and intended use of the software. Software becomes a medical device when the intention and function are to diagnose, cure, treat, prevent, or mitigate a specific disease or condition [28].

Should the app require premarket notification, multiple steps must be taken to satisfy the complex risk management strategies and regulatory requirements set by the FDA to release the app publicly. Rigorous clinical testing must be conducted to ensure that the device performance (1) is in line with the claimed performance and use case and (2) is similar in performance to the most analogous device currently approved and on the market (the claimed predicate device that must be identified for FDA submission). In addition to performance, the app must apply appropriate human factors and usability engineering processes, which must be assessed in standalone usability studies with at least 15 users in the desired user population [29]. Finally, a quality management system (QMS) must be in place to ensure that procedures and processes enable a reliable, safe, and effective software tool [30]. The QMS begins with the leadership and management plan to ensure that relevant stakeholders manage the medical app throughout its lifecycle. The support plans for the system must also be defined, with appropriate documentation of all development and design, disaster recovery plans, and processes for regularly updating the app for performance, usability, cybersecurity, and technology modernization. The implementation must be then considered, with regular validation, cybersecurity monitoring, and maintenance of all key infrastructure, to ensure that end users can interact with a stable version of the medical app throughout the duration of its lifecycle as a medical device.

#### Design strategy

**Fig. 4:**
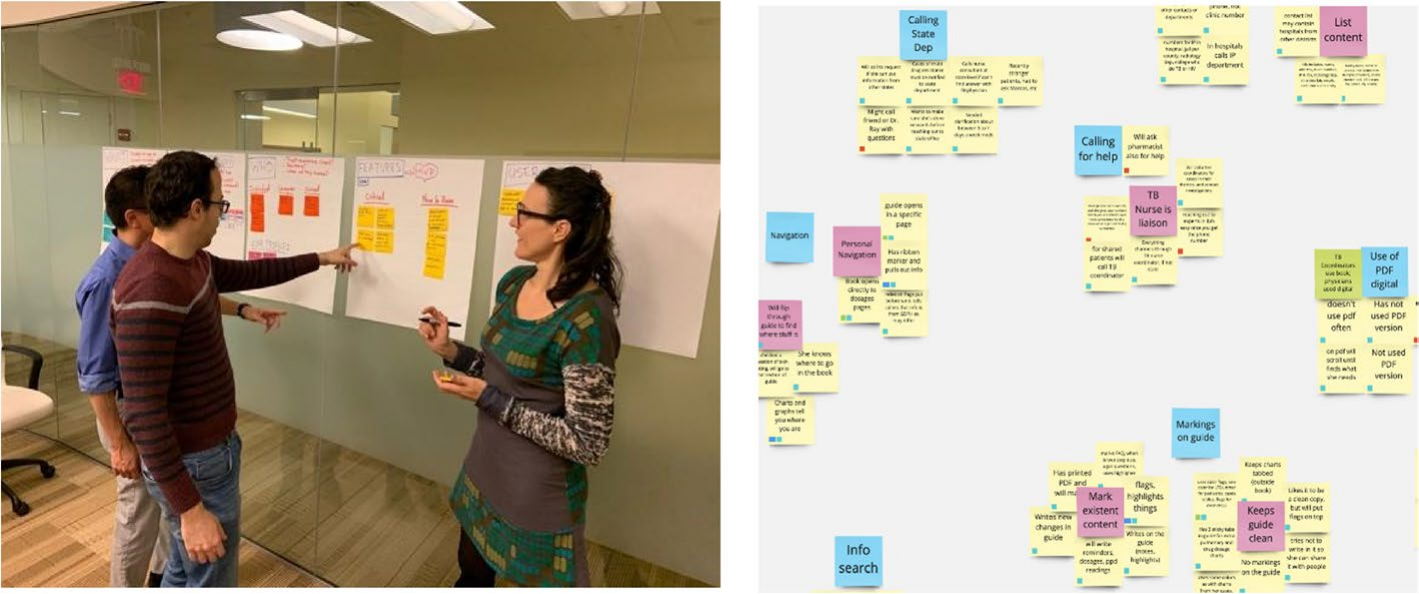
Collaborative design strategy discussions set the parameters of the project. Left: a design strategy session in person. Right: results of a design strategy session using an online collaboration tool like Miro

Once engaged on a project, a development team should schedule a design strategy session with the investigator and project team to gain a full understanding of the desired goal of the app from the perspective of all stakeholders (Fig. 4). While investigators often have a passionate idea about what problem they wish to solve with their app, additional examination can help dial into precisely who will use their tool and what problems their app user(s) seek to solve. To prepare for this session, which is usually 1–2 h, the team conducts secondary research from published academic papers, review articles, and meta-analyses to familiarize themselves with the outcome goals of the project and specific aims of the research. The team generates clarifying questions for the investigator and research team to respond to. Sample questions may include the following:

- Who will use this app? Is the patient or a family member the primary user?
- What benefit will the user get out of using this app?
- What data (if any) do you expect the app to collect?

Post-It notes or a collaborative whiteboard digital tool such as Miro (Miro, Inc., San Francisco, CA) may be used to capture ideas that can be sorted and prioritized as seen in the image below. This allows the team to gather insights from the session to form a user research approach to test assumptions, write and vet a discussion guide, and then recruit 5–15 individuals from the intended user group to interview. Insights across all interviews are pooled together and sorted to surface themes and areas of importance for the user. As the themes and key features emerge from the insights, the team can refine the insights into design recommendations.

#### App design

**Fig. 5:**
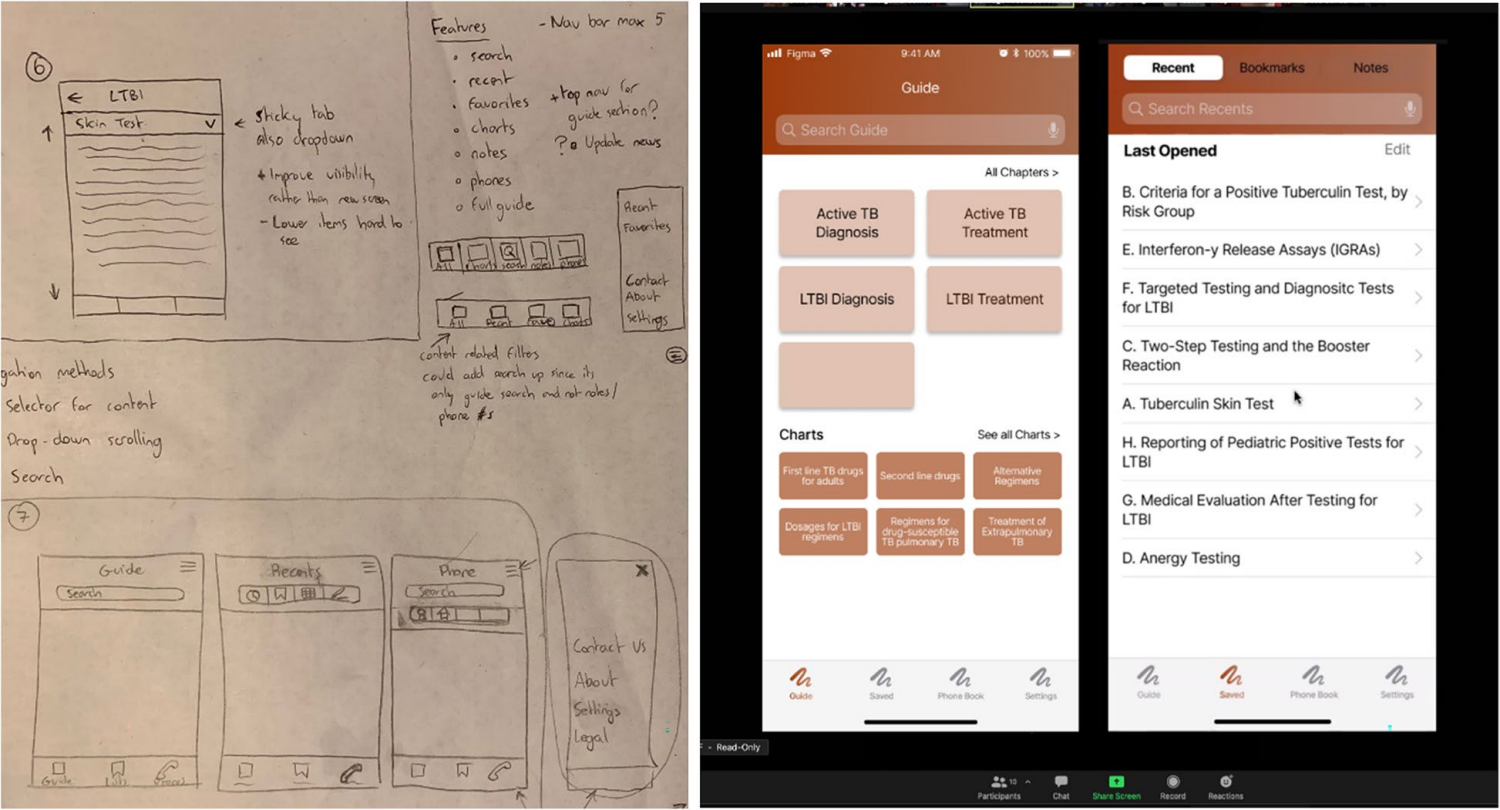
From sketches to wireframes. Left: pictures of preliminary sketches of the layout of the application. Right: functional (clickable) wireframes of an app

Once the research team has a clear idea of the needs and wants of the users, designs for the app should be created (Fig. 5). It is very important that these designs come from actual user insights, rather than from pre-conceptions the research team had prior to beginning the work, unless these pre-conceptions had been validated by the user research.

Given the number of apps that exist already, it is worth spending some effort looking to find similar apps that incorporate design patterns that could be implemented in the app. This is a separate step from the competitive analysis where rather than looking at apps that perform a similar function, you are looking for individual features (i.e., how search functions, how do other apps offer content layout).

Taking from the design recommendations generated in the prior phase. The designer can begin creating low-fidelity sketches, which they can turn into generalized flows and wireframes of the app (Fig. 5, left). The key is the use of wireframing and prototyping as an early way to evaluate whether the tool being created meets users’ expectations. To evaluate those wireframes, standard usability testing techniques can be used such as remote or in-person unmoderated observation sessions or task-based evaluations where the user is given tasks within the app to accomplish.

#### App development

The most common app development methodologies are the waterfall and agile methods [31]. The agile methodology is an iterative approach that leverages frequent releases that provide incremental functionality improvements that can be tested and refined in the production environment. The waterfall methodology completes the holistic build which then moves into a testing phase and then a launch phase, yielding a polished product. We prefer to utilize the agile methodology as the iterative development process enables more testing and feedback, which matches our design philosophy.

When an app development methodology has been selected, there are 2 major mobile app development frameworks: natively on each of the major operating system software tools (iOS or Android), or cross-platform (i.e., using React Native (Meta Platforms Inc., Menlo Park, CA), Flutter (Google LLC, Mountain View, CA), etc.) which supports one code base for both iOS and Android platforms (Table 1). The decision on what to use will largely depend on the expertise of the available developers in the team. This is the fundamental choice an app developer must make before developing an app.

**Table 1:** Overview of the development styles and platforms available

	**Native development**	**Cross-platform development**
iOS Devices	XCode (requires a Mac)	ReactNative Flutter etc
Android Devices	Android Studio	

Native development has the advantages of dedicated functions and features specific to the hardware you are developing for (iOS vs Android), with the downside that effort must be duplicated to support both iOS and Android devices. Also, native development tends to be more well-maintained, as it is linked directly to the mobile devices operating system. This skillset is also much more readily available in the software developer talent pool. While cross-platform development is typically much faster and more efficient with respect to developer resources, it also relies on a much smaller developer community. Furthermore, many 3rd party tools common in app development (e.g., analytics, advertisements) have limited support for cross-platform development. The development framework should be chosen to suit the needs of the specific project.

Throughout the development process, it is generally good practice to beta test the app to ensure that what is being built is meeting the user’s expectations. To do this, it is best to test in a controlled environment to monitor who is using your app. To do this you will use either TestFlight for iOS (Apple In., Cupertino, CA) or Google Play Store Internal Testing for Android (Google LLC, Mountain View, CA).

#### Launch

Once the app is launched, it must be closely monitored for bugs, unforeseen issues for users, and ever-increasing software versions. Both iOS and Android release beta versions of the updates in advance of a public release so that developers have a chance to test the compatibility of their apps against these updates. Nevertheless, there are many apps that are no longer supported by new OS versions that plague the app stores, a situation that should certainly be avoided by a medical app developer! Some thought should be put towards maintaining support for the users that may not have updated to newer operating system (OS) versions. It is typical to set a supported version that dates back at least a few years to give users time to upgrade their devices.

#### Updates and maintenance

Finally, app maintenance must be considered. Funds must be allocated to support the continuity of the app development project before it begins. As described by Siegel et al. [10], there are a multitude of unsupported apps on the app stores that are born out of research projects and then abandoned, likely due to the lack of personnel to support them, lack of funds, or poor design and execution leading to a lack of traction to justify a larger time/monetary investment.

Simultaneously with the design thinking process, it is important to work with the PI to help design a research study and secure IRB approval for the study protocol that will be used to clinically validate the app upon release. This validation study typically occurs in 2 phases: Phase 1 is focused on usability and feasibility. In this phase, user feedback of the app is collected and used to iteratively improve the app. This can be done by identifying users to enroll in the clinical study and conducting a rolling enrollment process where a version of the app is developed and given to the users to collect their feedback, adjust the app, and then repeat. Phase 2 is a clinical trial where the app’s performance is analyzed in terms of a clinical endpoint. In this phase, a randomly controlled trial should be planned to assess the app in terms of a clinical endpoint of interest, i.e., a control (no app) and test (has the app). These results can then be published in a medical journal to provide credibility to the app as a medical tool.

### Lessons learned: roadblocks, pivots, and best practices

#### App development costs

Funding for academic biomedical mobile apps can be difficult in our experience. The most common source of funding is through traditional National Institutes of Health (NIH) grants. These grants allow for setting aside resources in the budget to support mobile app development but do have some pitfalls. First, grants typically have a very long application and funding cycle, in the order of 12–18 months, which can delay a project. Second, funding amounts set aside for development tend to be small. In our experience, this has been $10,000–25,000 which is insufficient for the necessary costs associated with designing and developing an application (Tables 2 and 3). Beyond grants, academic medical centers often offer access to other seed or startup funds in the form of innovation awards, local organizations that offer awards or seed funds, and, as a last resort, projects can be funded through department funds if the funds and scope of the project are aligned.

**Table 2:** Personnel costs for mobile app development

Staff cost	Per hour estimated cost
Graduate student intern	$30/h
Outsourced app developer (India)	$30–50/h
Academic app developer	$60–100/h
App development consultants	$100–250/h
Experienced designer	$60–100/h

**Table 3:** App development cost breakdown by stage

Mobile app development stage	Estimated cost
User and market research	$2000–10,000
User interface design	$5000–10,000
Software development	$10,000–50,000
Software maintenance (annual)	$1000–5000

Staffing an academic medical center app development project is very different from a commercial software development project. The key differences are (1) the budget of the project is typically far lower (by an order of magnitude, typically), (2) the goal of the app is often non-commercial (e.g., it does not have a business model attached), and (3) the goals of the PI may include education and training for the full team, beyond only executing on the creation of the software. Therefore, staffing for mobile app projects will use a mix of resources available within academic medical centers and their affiliates. These include graduate or undergraduate students who may work on a project for free as part of a classroom learning experience, students who are hired outright or work through graduate research assistant positions, shared staff with other programs (e.g., bioinformatics), and partnership with other campus-based groups who are funded to support clinical research.

#### Managing investigator expectations

We often encounter requests that are in essence to “just build an app” from clinical PIs. It is important to diagnose this way of thinking early as it could reveal the propensity of a PI to disengage from the project prematurely, as it becomes clear the amount of effort and collaboration it takes to foster a successful partnership. PIs must be engaged often and richly with the team. The cadence of meetings will be project dependent, but we have found a weekly update, whether a meeting or email, maintain project members active and engaged.

Having a disengaged PI can lead to multiple points where the project can fail: (1) the problem is not defined appropriately, (2) the appropriate user research is not conducted (which can lead to the app not addressing all relevant user needs thus impacting adoption and use), and (3) usability/feasibility testing is not conducted in a robust and iterative fashion (which can lead to usability issues that will prevent users from adopting the app, even if the app has a strong value proposition that could significantly improve the life of the user). In addition, it taxes the rest of the team who must make clinical research decisions and navigate an unfamiliar research structure.

#### Understanding the users

It can be challenging to identify and access users to solicit feedback from. Clinician investigators typically have frequent interactions with the target user population. They have familiarity and a relationship built already (which is how the ideas for projects usually originate). However, they usually lack the time to conduct thorough user research that goes beyond the problem discovery. On the other hand, developers and designers do have the time and knowledge to conduct user research, albeit they lack access to patients. Bridging this gap, often by facilitating access to clinics and hospitals for the design team, can drastically improve a project’s chance of success. In one of our projects with Grady Memorial Hospital, we established a cadence of going to the hospital twice a week to enroll new subjects and check in on existing subjects. Furthermore, fostering a relationship with the clinical care teams of the subjects facilitates subject enrollment, a process that is much slower when only investigators are involved in subject recruitment. Additionally, by fostering that relationship with the clinical care of the subjects, it is possible to conduct user research with stakeholders through observation sessions and contextual inquiry. Which can help mitigate some of the overreliance on user input [32].

#### Beta testing recruitment

Recruitment can take place in person or virtually. Online recruitment tends to require less apparent overhead (although depending on the clinic setting it may be hard to capture patients online) while in-person recruitment has a higher overhead but tends to yield more numbers and more upfront engagement. Most of the recruitment that we have done has taken place in person at the hospital. Interestingly, we have found that subjects recruited in a clinical setting will remember everything about the app and the study until they leave the hospital/clinic, when study participation significantly wanes. To increase the chances of success, you must find the best method to follow up with your subject/users which could be via email, text, or phone calls so that you can maintain engagement with your study. This will be very study specific and dependent on the app you build. This is one area where sampling bias can impact the project as subjects who are more interested are more likely to respond and be engaged. That was our experience with that project, and we had very low compliance from the users we were trying to target, those who leave the hospital and do not return.

#### Beta testing process

Testing an app with users frequently is crucial to ensure the best possible version of the app is developed. In an in-patient setting, we have found it necessary to introduce users to the app in one session, and then follow up within a week, to see if the patient interacted with the app after the visit. There are multiple steps that can be taken to foster engagement:

- Delay the follow-up: A week might not be enough time for the user to have a need to open the app.
- Include push notifications: This system allows researchers to send messages through the app to specific segments of the study group (i.e., users who have not opened the app recently).

The goal of beta testing is to find encounter any bugs and errors and more importantly if the intended user audience finds the app useful. It is very challenging to design a research study surrounding a tool that will fit into a user’s life outside of the study. This is because study coordinators are actively looking for subjects to enroll, creating unnatural motivators to utilize the tool (i.e., participate in research, monetary compensation, curiosity), as opposed to an organic app search where users are looking for something. Thus, it is important to design an app such that the app fills a clearly defined and currently unmet need for a group of people (i.e., a medical reference guide, an appointment booking system, a prescribed training program). Otherwise, you run the risk of aiming to change a given behavior which has been identified as a limitation of traditional HCD and follows a different design process [32].

Furthermore, mobile apps have standardized evaluation frameworks such as the System Usability Scale (SUS) which provide a quantitative evaluation of the usability of a software tool. Note the difference between usability: Can somebody use the app without encountering errors that worsen their experience, and feasibility? Does the app effectively cover an unmet need for a user? Usability is an easier metric to quantify, and mobile apps for research should follow industry standards to validate the tools created are usable. These frameworks can be employed to glean valuable feedback during the beta testing process.

## Conclusions

Overall, we have developed and validated an organization within a clinical research institution that fostered communication between clinicians and app developers and enabled the creation of robust, sustainable medical apps that are currently available on the App Stores and being tested in clinical settings. This organization is the first of its kind and can be used as a blueprint for research institutions to use to facilitate the translation of medical app research and transform the digital health landscape worldwide.

## Data Availability

Not applicable.

## Abbreviations

EHR: Electronic health records
FHIR: Fast Healthcare Interoperability Resources
CTSA: Clinical and Translational Science Alliance
DT: Design thinking
HCD: Human-centered design
PI: Principal investigator
UX: User experience
HIPAA: Health Insurance Portability and Accountability Act of 1996
IRB: Institutional Review Board
PHI: Protected health information
US: United States
FDA: United States Food and Drug Administration
QMS: Quality management system
CA: California
OS: Operating system
NIH: National Institutes of Health
